# Automatic worm detection to solve overlapping problems using a convolutional neural network

**DOI:** 10.1038/s41598-022-12576-9

**Published:** 2022-05-20

**Authors:** Shinichiro Mori, Yasuhiko Tachibana, Michiyo Suzuki, Yoshinobu Harada

**Affiliations:** 1 Institute for Quantum Medical Science, National Institutes for Quantum Science and Technology, Inage-ku, Chiba, 263-8555 Japan; 2 Takasaki Advanced Radiation Research Institute, National Institutes for Quantum Science and Technology, Takasaki, Gunma 370-1292 Japan

**Keywords:** Molecular biology, Neuroscience, Mathematics and computing, Nanoscience and technology

## Abstract

The nematode *Caenorhabditis elegans* is a powerful experimental model to investigate vital functions of higher organisms. We recently established a novel method, named "pond assay for the sensory systems (PASS)”, that dramatically improves both the evaluation accuracy of sensory response of worms and the efficiency of experiments. This method uses many worms in numbers that are impractical to count manually. Although several automated detection systems have been introduced, detection of overlapped worms remains difficult. To overcome this problem, we developed an automated worm detection system based on a deep neural network (DNN). Our DNN was based on a “YOLOv4″ one-stage detector with one-class classification (OCC) and multi-class classification (MCC). The OCC defined a single class for worms, while the MCC defined four classes for the number of overlapped worms. For the training data, a total of 2000 model sub-images were prepared by manually drawing square worm bounding boxes from 150 images. To make simulated images, a total of 10–80 model images for each class were randomly selected and randomly placed on a simulated microscope field. A total of 19,000 training datasets and 1000 validation datasets with a ground-truth bounding-box were prepared. We evaluated detection accuracy using 150 images, which were different from the training data. Evaluation metrics were detection error, precision, recall, and average precision (AP). Precision values were 0.91 for both OCC and MCC. However, the recall value for MCC (= 0.93) was higher than that for OCC (= 0.79). The number of detection errors for OCC increased with increasing the ground truth; however, that for MCC was independent of the ground truth. AP values were 0.78 and 0.90 for the OCC and the MCC, respectively. Our worm detection system with MCC provided better detection accuracy for large numbers of worms with overlapping positions than that with the OCC.

## Introduction

The nematode *Caenorhabditis elegans* (*C. elegans*) is a powerful experimental tool for model building to investigate vital functions, i.e., stimulation responses (chemotaxis, thermotaxis, and mechano-sensation), motor control, developments, aging, learning and memory. Especially, their excellent sensory system that response to chemical substances (taste and smell) have been well studied^1–3^. The mechanism has been partially clarified based on the conventional chemotaxis assay method^4,5^. However, there was a major concern that the conventional method of chemotaxis assays using the excellent odor and taste sensitivity of *C. elegans* is not yet good accuracy, because it is a bit bother to count over 100 worms on the 10 cm diameter plate, some worms were gathered or out of the test substance area^4,5^. Recently our group established the novel method "pond assay for sensory system (PASS) method", and it dramatically improved both the evaluation accuracy of sensory response and the efficiency of experiments by counting worms on small ponds filled the test substance solution^6,7^.

Worms have simple cylindrical bodies (approximately 1 mm) and varied complex postures and motions (wave type, omega type, coil type)^8^, making them difficult to count manually, especially if they overlap. Therefore, shooting an image or immobilization with anesthetics is effective method to count individuals exactly. Thanks to significant progress in computer vision and machine learning, automated worm detection systems have been developed which achieve high throughput and increase detection accuracy^9–13^. Nagy et.al and Ochoa et.al. reported automated worm detection methods with overlapping object problem^12,13^. Bargmann et al. reported to place between 100 and 200 washed adult worms on a 10-cm chemotaxis assay plate^4^, and Hirotsu et al. reported to placed approximately 50–100 worms on the 10-cm chemotaxis assay plate^14^. Most of these have been applied to small numbers of worms, low worm population density, and/or did not evaluate overlapped worms or complex postures. As we know, there are no reports to detect worms over 100 individuals under the crowded condition in small pond, especially in PASS method.

To overcome this problem, we develop an automated worm detection system which deals with worm overlapping. Our worm detection system uses multi-class classification (MCC) for worms with a deep neural network (DNN). Here, we describe the technical aspects of the worm detection system and compare detection accuracy between one-class classification (OCC) and MCC.

## Results

**Figure 1:**
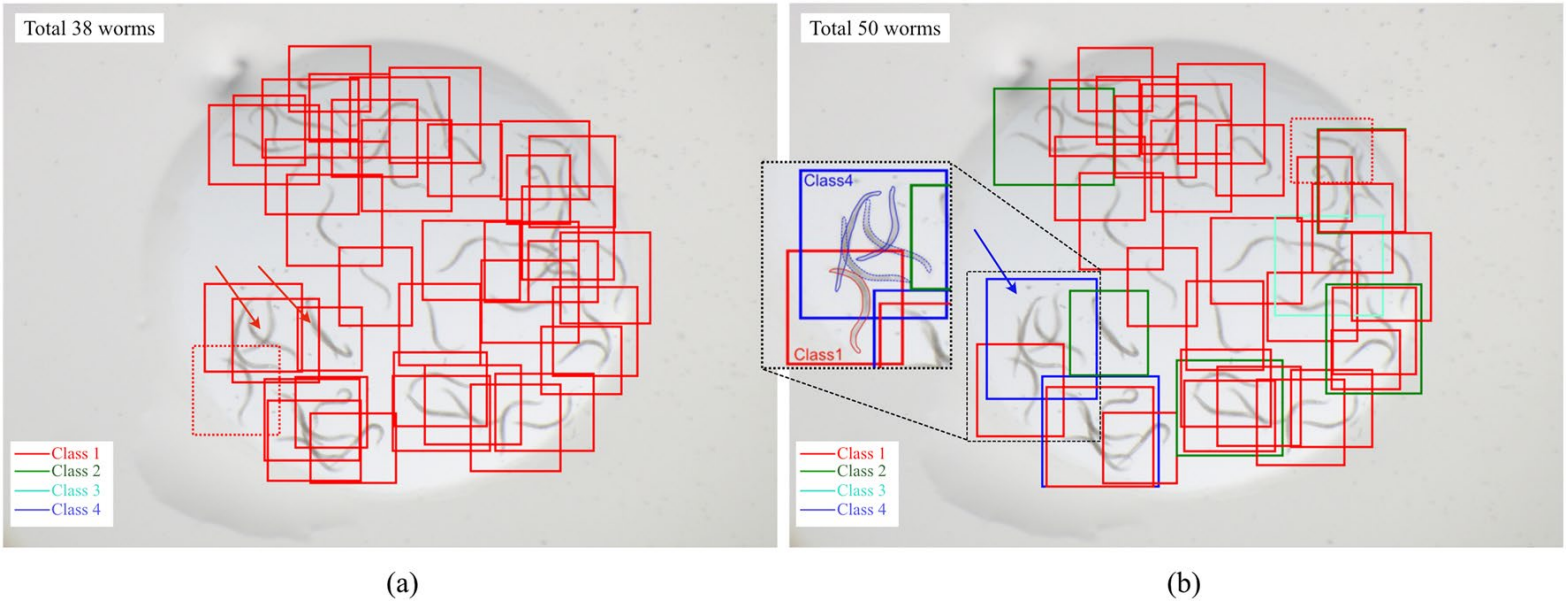
Results for the worm detections with (**a**) the one-class classification (OCC) and (**b**) the multi-class classification (MCC). Red, green, light blue, and blue bounding boxes show Class 1, Class 2, Class 3, and Class 4, respectively. The ground truth number of the worms was 46.

One example of detection with the OCC and the MCC is shown in Fig. 1. The correct number of worms was 46. The OCC detected 38 worms of variable sizes and shapes, and in cases of a few overlapping worms, at most two worms were detected. However, some overlapped cases were not detected (marked with red arrows in Fig. 1a). In contrast, the MCC detected 50 worms and 2, 3, and 4 overlapped worms correctly in all but one case, detecting five worms as Class 4 × 1 + Class1 × 1 even though Class 5 was not set (marked as blue arrow in Fig. 1b). The bounding boxes in Fig. 1b with the same red arrows as in Fig. 1a were detected correctly.

From all evaluation data, a total number of bounding boxes detected for OCC and MCC were 4301 and 3722, respectively. And then, that for MCC was 5331 by dividing Class *n* into Class 1 × *n*. Precision values were 0.91 for both OCC and MCC. The recall value for MCC (= 0.93) was, however, higher than that for OCC (= 0.79). From these results, F1 values were derived as 0.85 and 0.92 for OCC and MCC, respectively.

**Figure 2:**
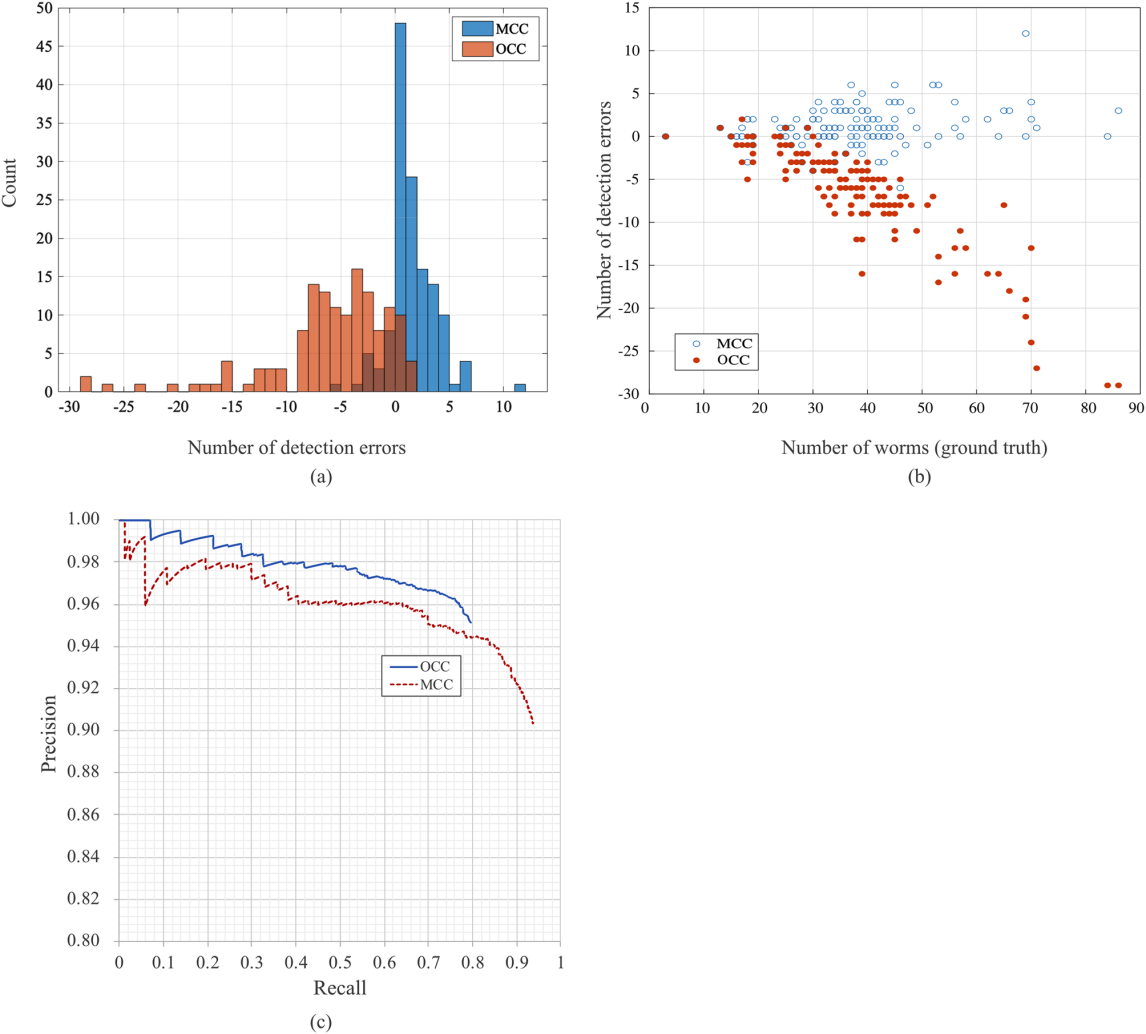
(**a**) Histogram for the number of detection errors in OCC and MCC. (**b**) Scatter plot shows the number of worms (ground truth) and the number of detection errors for OCC and MCC. (**c**) Precision-recall curve for the OCC (solid line) and MCC (dotted line). IoU threshold is 0.5. *Abbreviations: OCC* = *one-class classification, MCC* = *multi-class classification, IoU* = *Intersection over union*.

The number of detection errors was calculated by the number of detections minus ground truth. These results are summarized as a histogram in Fig. 2a. The average detection error (average ± standard deviation) was −6.3 ± 5.7 and 1.0 ± 2.2 for OCC and MCC, respectively. Regarding the OCC, the number of zero detection errors was 10, and the maximum number of detection errors was 29. Most of the detection errors were caused by negative values, indicating underestimation by the OCC. Most of the detection errors for MCC showed ± 5 worms. MCC has a lower detection error compared to OCC.

To understand the relationship between the object detection error and the number of objects (ground truth), we summarized our results as a scatter plot in Fig. 2b. The number of detection errors for OCC increased (negative value) with increasing ground truth. More than 70 ground truth objects were not counted over 25 objects. The number of detection errors for MCC, however, was independent of the ground truth.

To evaluate the relationship between recall and precision quantitatively, precision-recall curves for both OCC and MCC were calculated with an IoU threshold of 0.5 (Fig. 2c). The two curves were similar, but the precision value of the MCC was higher than that of the OCC over a recall of 0.6. Average precision values were 0.78 and 0.90 for the OCC and MCC, respectively. These results are summarized in Table 1.

**Table 1:** Results of the detection rate metrics (precision, recall, average precision, and a total number of detected bounding boxes) with OCC and the MCC. *Abbreviations: OCC* = *one-class classification, MCC* = *multi-class classification*.

	OCC	MCC
Precision	0.91	0.91
Recall	0.79	0.93
F1	0.85	0.92
Average precision	0.78	0.90
A total number of detected bounding boxes	4301	3722

## Discussion

We developed a new DNN for detecting overlapping and complexly arranged worms and compared the detection accuracy between the OCC and the MCC. Mean precision was 0.97 and 0.80 for the MCC and OCC, respectively. The MCC detected overlapped worms better than the OCC. The AP value for the MCC, however, showed sufficient accuracy to detect worms in our situation. When larger numbers of worms are overlapped, the number of class labels might need to be increased.

**Figure 3:**
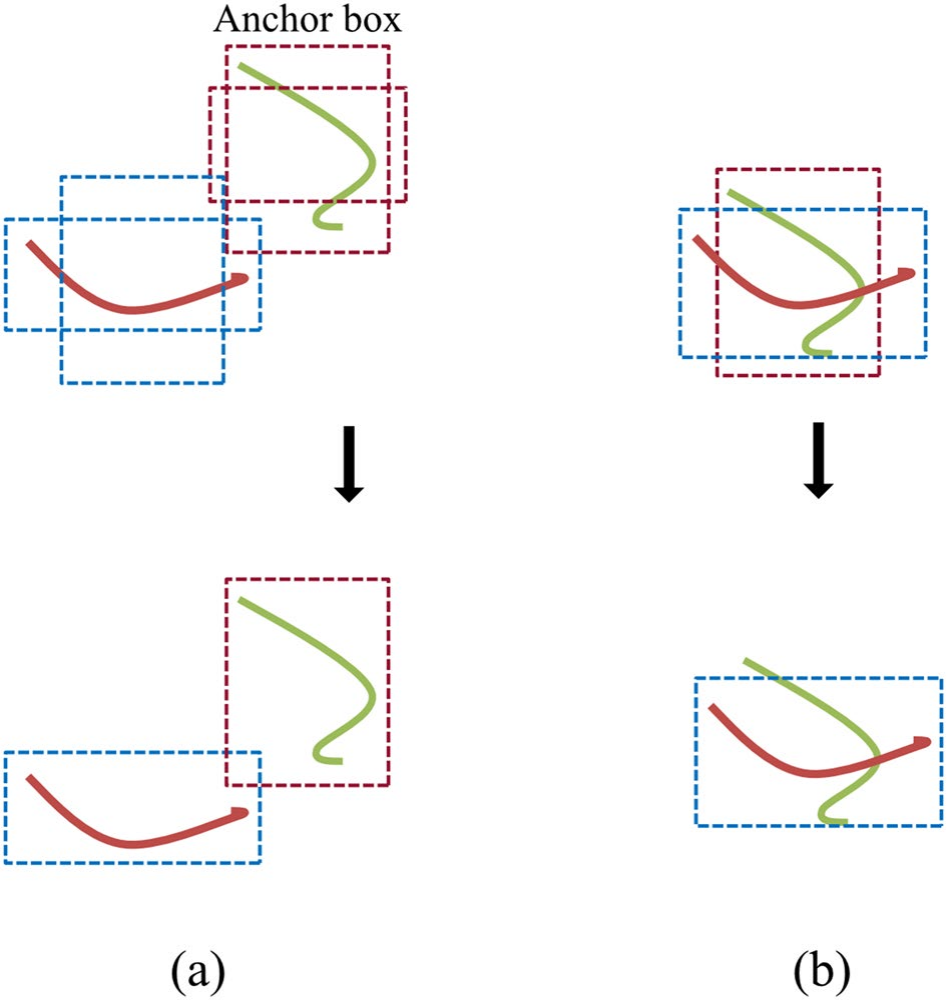
Example of anchor box selection for two objects. The dotted rectangles are anchor boxes over the confidence score threshold. The red and green curves show objects. (**a**) Non-overlapped objects case. One anchor box for each object is selected from two anchor boxes for each object by NMS. (**b**) Overlapped objects case. One anchor box is selected from two anchor boxes by NMS due to close locations less than IoU threshold. *Abbreviations: IoU* = *intersection-over-union, NMS* = *non-maximum suppression*.

The main reason for incorrect detection of overlapped worms was due to the non-maximum suppression (NMS) algorithm, as described in Materials and Methods (Fig. 3). Several publications have introduced modified NMS; however, NMS performance is dependent on circumstances^15–17^. Other studies have introduced a neural network-based duplicate data removal method^18,19^. These approaches can detect common objects such as cars, dogs, humans, etc., and overlapping objects are included in the occluded region. Although worms have varied and complex postures and the occluded area of overlapping worms is small, these characteristics are very different from those of common objects. We used the MCC to solve the overlapping object problem for worm detection; this approach did not modify the DNN algorithm but rather simply increased the number of class labels.

Regarding to the detection accuracy, the OCC and MCC shared the same precision value (= 0.91). The background of the original image was almost uniform and the number of object types was 1; therefore, DNN could detect any objects as worms, resulting in the same precision value. However, precision does not consider false-positives; that is, it does not evaluate misdetection, especially in overlapped worms. Recall considered the false-positive rate and expressed important performance metrics in our study. Recall values for the MCC were higher than those for the OCC; therefore, the MCC detected overlapped worms more accurately. Our DNN’s AP was 0.9, which is higher than that in other studies^20–22^. The latest DNN, which used MCC for different types of objects, achieved an AP of 0.3–0.5 for the Microsoft Common Objects in Context dataset^20,21,23,24^. This is because our DNN was developed to detect worms only.

Approximately 30% of all premature deaths (= 15.2 million) from noncommunicable diseases in 2016 were due to cancer^25^. It is well known that cancer screening, for detection of early disease in specific cancers, has resulted in substantial declines in cancer mortality^26^. In 2015, it was reported that the nematode *C. elegans* are drawn to the urine of cancer patients (chemotaxis) and can be applied to cancer screening test^14^. Middle- and high-income countries focus more on preventive medicine, cancer screening test using *C. elegans* will be employed in the world in near future. The proposed worm detection method will become an important tool to accelerate full automation of the cancer screening test using *C. elegans*.

A few limitations of this study warrant mention. First, we set four classes for MCC and did not evaluate the number of class dependencies. Allowing that an optimum number of classes might improve the detection rate, our study clearly showed that had MCC improved detectability for overlapped worms compared with OCC. We plan to optimize the number of classes in our next study. Second, the main topic of our study was to detect worms by applying the MCC, and we did not compare detection accuracy using other DNNs While the AP values of the EfficientNet and YOLOv4 are almost the same, the detection speed of the YOLOv4 is approximately twice as fast. When new DNNs with higher accuracy are developed, we will reevaluate worm detectability.

## Conclusion

Our worm detection system with MCC provided better detection accuracy for many worms with overlapped postures than that with OCC. Use of our system would be widely used to assays using worms and may eventually improve throughput.

## Methods

### Strain and culture

*C. elegans* wild-type (N2) strains and *Escherichia coli* strains OP50 were obtained from the Caenorhabditis Genetics Center (The University of Minnesota, Minneapolis, MN, USA). *C. elegans* hermaphrodites were grown at 20 °C on a 10-cm plate (IWAKI nontreated dish; AGC Techno Glass, Shizuoka, Japan) containing 20 mL of nematode growth medium (NGM) spread with a bacterial lawn as food^27^. Well-fed adult worms, approximately three days after hatching, were used in experiments. Worms were cultivated in synchronization to make the individual stage uniform, however, it is not required to fix the worm size in this study.

### Sample preparation for worm detection

*Caenorhabditis elegans* individuals were collected from the culture plate using a gelatin-based wash buffer solution (containing 5 mL of 1 M potassium phosphate (pH 6.0), 1 mL of 1 M CaCl_2_, 1 mL of 1 M MgSO_4_, and 0.5 g gelatin in 1 L of H_2_O; sterilized by autoclaving), and washed twice with a wash buffer solution^5^. Two or four small recesses (5 mm in diameter and approximately 2 mm in depth) for ponds on a plate were formed on an assay agar plate and fulfilled it with ~ 30 µL of saline^6,7^. Approximately 10–100 washed worms were dropped into each small pond and covered the plate with a plastic lid.

### Image acquisition

The pond was photographed using a digital camera (High-Speed EXILIM, Casio Computer Co., Ltd., Tokyo, Japan) mounted on a fluorescence stereomicroscope (SZX16, Olympus Corporation, Tokyo, Japan) with the objective lens (× 1) (SDFPLAPO1 × PF, Olympus Corporation, Tokyo, Japan). A total of 300 different pond photographs (RGB 8-bit color depth, original images) were acquired with an image size of 2816 × 2112 pixels.

### Image pre-processing

**Figure 4:**
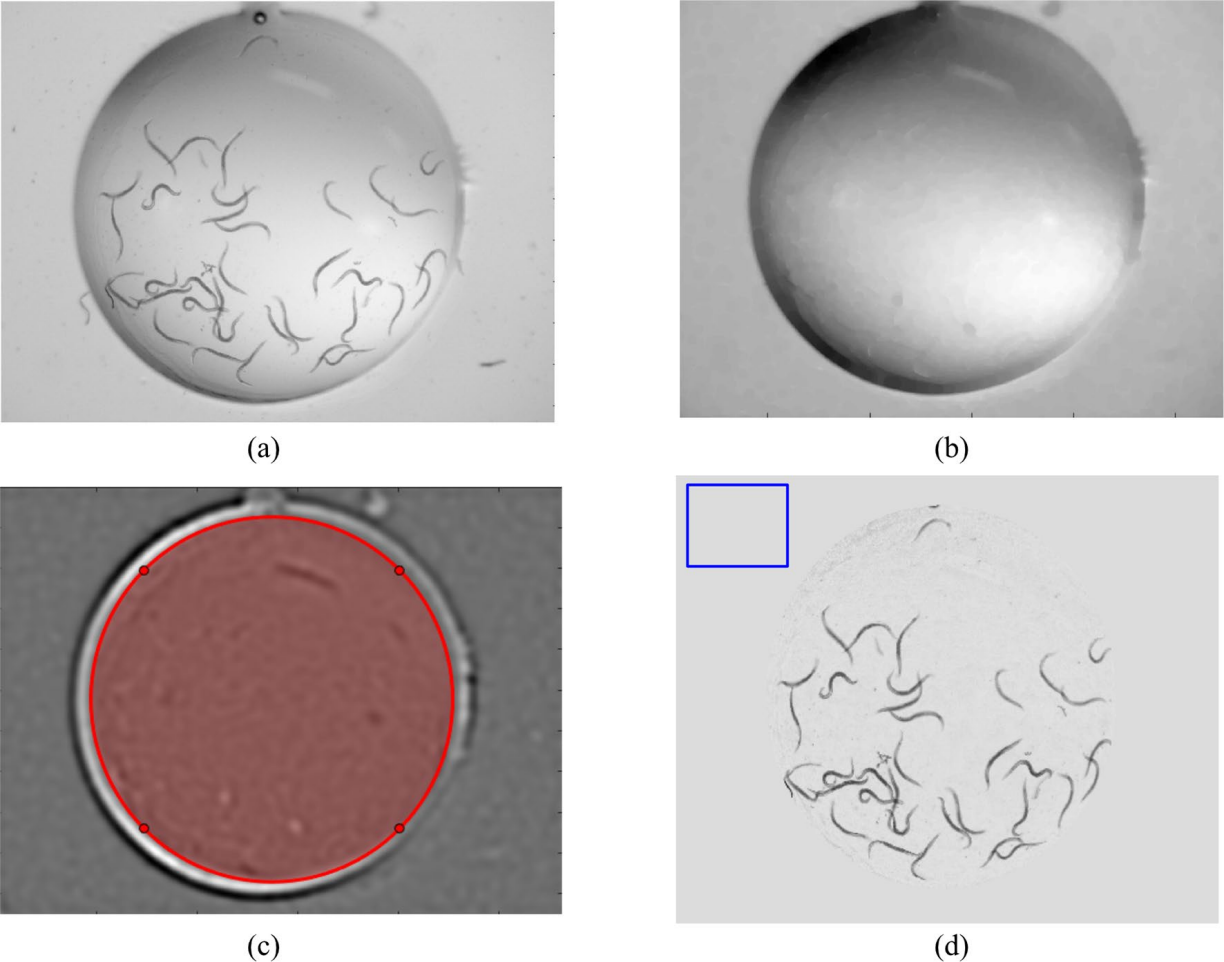
Image background correction processing. (**a**) Resized original image (image a). (**b**) The image process “closing” and a median filter were applied to Image a, resulting in Image b. (**c**) Image processing of difference of Gaussians (DoG) was applied to Image b, resulting in image c). The detected hole edge was marked as a red circle. (**d**) Background corrected image (image d).

The original image dimension of 2816 × 2112 pixels was resized to 704 × 528 pixels. Images with RGB 8-bit depth were converted to grayscale (Image A, Fig. 4a). Since the nonuniform contrast of the background and the hole edge curve could be affected by the object detection accuracy, we applied background correction as follows:

(i) The image process “closing,” which removes disk shapes (= 20–30 pixel diameter, and a median filter were applied to Image A, to generate a background correction image (Image B, Fig. 4b)^28^.
(ii) To emphasize the pond edge, a difference of Gaussians (DoG) was applied to Image B; that is, a Gaussian blurred with a kernel size 20 × 20 pixels of Image B was subtracted from a Gaussian blurred with a kernel size 30 × 30 pixels of Image B (Image C, Fig. 4c)^29^.
(iii) The pond edge of Image C was detected by the Circle Hough Transform (marked as a red circle in Fig. 4c)^30^
(iv) Finally, Image D was generated the pixelwise division of Image A by Image B, and the mean pixel value of Image D was calculated around the periphery of Image D (e.g., marked as a blue square in Fig. 4d). The mean value was filled in on Image D outside of the detected pond edge (Fig. 4d).

### Network architecture

**Figure 5:**
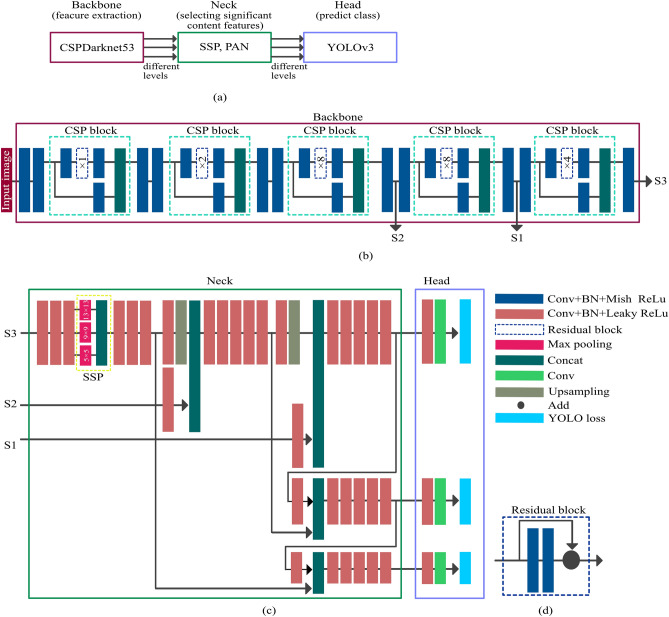
(**a**) Deep neural network structures, which are composed of three major parts (Backbone, Neck, and Head). (**b**) Network structures for Backbone, which contains 52 convolutional layers with CSP block to reduce dimensions. The number of repetitions for the residual block is in the blue dotted rectangle. (**c**) Network structures for Neck and Head. Input data (S1, S2, and S3) is from the Backbone (S1, S2, and S3). (**d**) The residual block, which applies shortcut connections to involve two sets of convolutional (Conv), batch normalization (BN) and ReLU layers. *Abbreviations: CSP* = *Cross stage partial, SSP* = *Spatial pyramid pooling, PAN* = *Path aggregation network, ReLU* = *Rectified linear units, YOLO* = *you only look once.*

Several publications have introduced DNNs for object detection for single and/or multiple objects^20–23,31–33^. These DNNs predict object position and category by using region information (= bounding boxes). We used a “you only look once (YOLOv4)” method to detect worms in our study. Our DNN for worm detection was composed of three major parts: Backbone, Neck, and Head (Fig. 5a).

Backbones derive features from input images and detect multiple small objects by deriving the representation with reduced spatial dimensions. Backbones are based on CSPDarknet53, which is based on Darknet53 (containing 52 convolutional layers) but replaces the residual block with the Cross stage partial (CSP) block (Fig. 5b). Two sets of convolution (Conv), batch normalization (BN), and layers composed of Mish rectified linear units (ReLU), ReLU, and the CSP block were repeated five times. Conv + BN + Mish-ReLU layers were then added^34,35^. CSP block separates the feature map of the base layer into two parts. One part (half of the feature channels) contains Conv + BN + Mish-ReLU layers, residual block, and Conv + BN + Mish-ReLU layers, and goes through a residual block and a transition layer. The second part contains Conv + BN + Mish-ReLU layers, and is then combined with the transmitted feature map. The residual block applies shortcut connections to involve two sets of Conv + BN + Mish-ReLU layers (Fig. 5d).

The Neck selects significant content features from Backbones using Spatial Pyramid Pooling (SPP) and a Path Aggregation Network (PAN)^36,37^ (Fig. 5c). Three input datasets are imported from Backbone (S1, S2, and S3) and applied to multiple sets of Conv + BN + Leaky ReLU layers. After three sets of layers ([Conv + BN + Leaky ReLU] + [Conv + BN + Leaky ReLU] + [Conv + BN + Leaky ReLU]) are connected, the input datasets (S3) are applied to SSP. SSP is a special pooling layer using three different filter sizes (5 × 5, 9 × 9, and 13 × 13 pixels) to export a constant dimension (marked as the yellow dotted line in Fig. 5c). PAN boosts the feature information through the different spatial dimensions.

Heads predict classes and object locations from the bounding box using YOLOv3 (one-stage)^23^ (Fig. 5c). The filter size of the convolutional layer before the YOLO layer was calculated by

1 $$(the\ number\ of\ classes + 5) \times 3$$

The resulting filter sizes for the OCC and the MCC were 18 × 18 and 27 × 27, respectively. To achieve fast and accurate prediction of the object location and size, YOLO layered tiled anchor boxes across the image, and calculated the joint probabilities for the respective anchor boxes. Finally, the object class and location (i.e., the bounding boxes) were derived by selecting the high/top-scored anchors.

These tasks were performed using three different dimensions, one each for Backbone, Neck, and Head parts.

### Network training

#### OCC and MCC

In object detection, a class label is assigned to each object. Since our DNN detects worm only, a single class label is generally assigned (OCC). However, detection of overlapping objects remains difficult, for the following reasons:

As an example of two non-overlapping objects, anchor boxes (two in this case) are selected using threshold over the confidence score, which is the probability of the prediction accuracy (upper panel in Fig. 3a). The final anchor box over the confidence score threshold for each object is selected using non-maximum suppression (NMS) (upper panel in Fig. 3b)^33^. NMS rejects anchor boxes for each class if they display intersection-over-union (IoU), which is defined as the result of dividing the area of overlap between the bounding boxes by the area of union, the overlap with anchor box being higher than a learned threshold. Since the IoU value for the final anchor boxes is less than the IoU threshold, both anchor boxes are separated.

The next example is the overlapped objects. Anchor boxes over a greater confidence score threshold are obtained, but they are very close and often overlap. NMS selects one anchor box using the IoU threshold (lower panel in Fig. 3b).

To solve this problem, we used an MCC to assign class labels for respective numbers of objects. By doing this, overlapped objects could be detected as a multi object class. In this study, we set four classes according to observation of the original images. This is explained as following: Assuming that the worm length is approximately 1 mm, a 0.7 mm square with the fully extended state as the diagonal (1 mm) could correspond to the range of worm movement (= 0.49 mm^2^) when the worm varied complex postures. The pond area is 19.625 mm^2^ (5 mm-diameter). Then, the maximum number of the worms without overlapping can be derived approximately 40 worms (= 19.625 mm^2^/0.49 mm^2^). Since we dropped 100 worms at the maximum on the PASS plate^7^ with two or four ponds in our study, the number of worms dropped into one pond is 100 at most. Therefore, we estimated the number of overlapped worms is approximately 3 worms (> = 100 worms / 40 worms).

#### Training data

**Figure 6:**
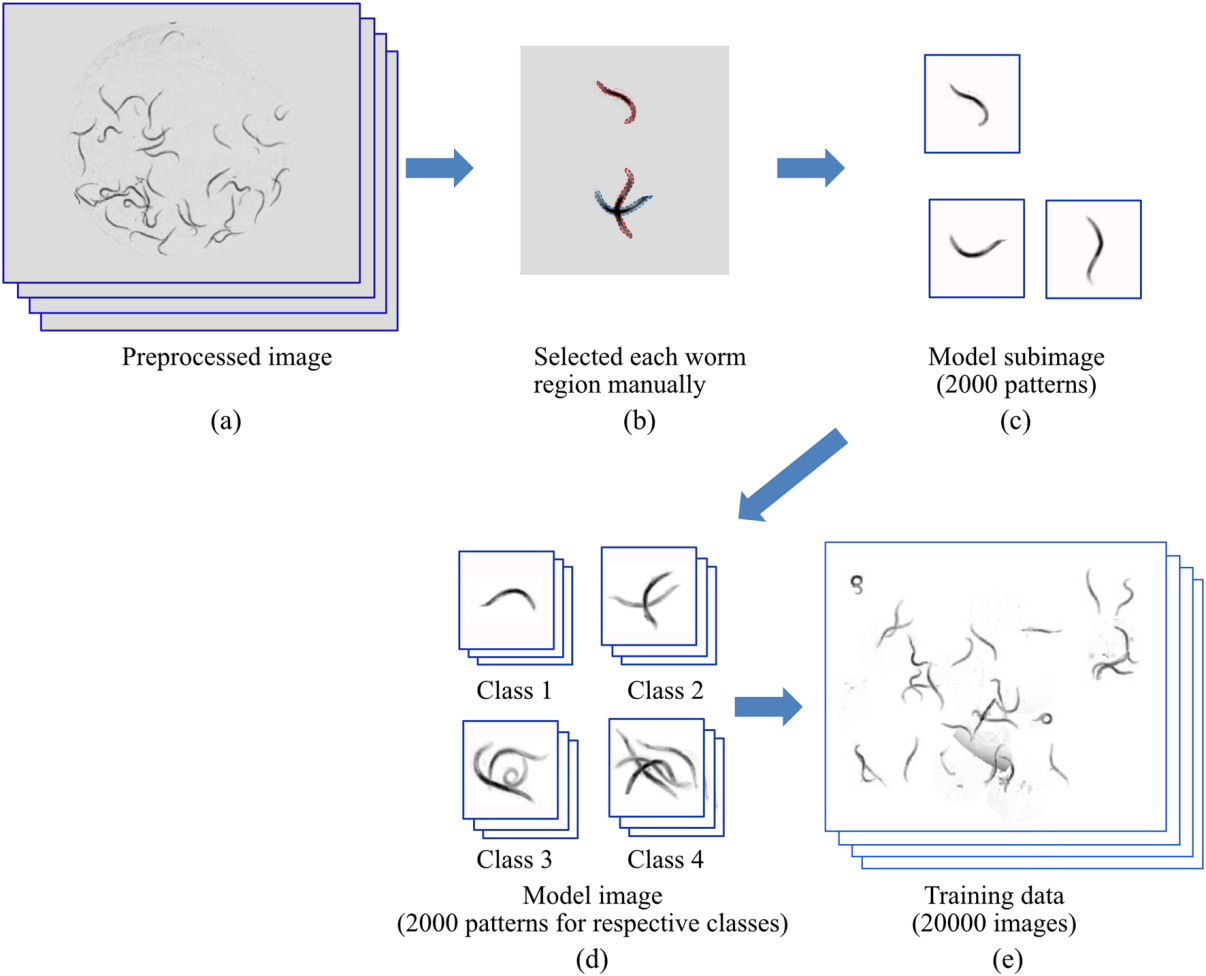
(**a**) Preprocessed image, which underwent background correction. (**b**) Each worm region was selected manually to separate one worm. (**c**) Worm model sub-image, which was manually selected from the preprocessed images. (**d**) Model images with a single, overlapping 2, 3, and 4 worms for respective Class 1, Class 2, Class 3, and Class 4 by selecting the model sub-image randomly. (**e**) Training dataset.

Generally, a large amount of training data is required to improve detection accuracy; however, it takes a long time to input the worm positions, which are defined with bounding-boxes (left top X and Y positions, width and height) (= ground truth). We prepared the worm model images training data using the following steps:

(i) We randomly selected 150 images from the 300 original images, and used the other 150 images for evaluation.
(ii) The background correction described in the previous section was applied to the images (Fig. 6a).
(iii) Each worm region was selected manually to separate to one worm except when more than 3 worms were overlapping (Fig. 6b). A total of 2000 patterns of model sub-images (100 × 100 pixels) were prepared (Fig. 6c). The ground-truth bounding-boxes were also defined.
(iv) For the MCC, we prepared model images with single and 2–4 overlapping worms for Class 1, Class 2, Class 3 and Class 4, respectively, by selecting the model sub-image randomly and applying rotation and/or zoom randomly (Fig. 6d). The ground-truth bounding-box for Class 1 (Model image, Fig. 6d) was the same as that of the model sub-image (Fig. 6c). The ground-truth bounding-box for Class 2–Class 4 was defined to encompass the ground-truth bounding-box for respective model sub-images. For the one-class classification, the ground-truth bounding-box for the model image with overlapping worms (Class 2–Class 4) was defined by respective model sub-image positions.
(v) A total of 10–80 model images (Class 1 - Class 4) were randomly selected and randomly placed on a simulated image field. As a result, a total of 19,000 training datasets and 1000 validation datasets were prepared with the ground-truth bounding-box (Fig. 6e).

All image processing for the training data was performed using commercial software (MATLAB R2020a, Mathworks, Natick MA, USA).

#### Parameter optimization

An optimization procedure was performed with 37,000 iterations with a batch size of 3 using stochastic gradient descent (SGD) to minimize the regression loss (Complete IoU (CIoU) loss)^38^ between the output bounding-box through the DNN and the ground-truth bounding-box. The learning rate, weight decay, and momentum were 0.001 (multiple 0.8 in each 50,000 steps), 0.0005, and 0.949, respectively. The deep learning framework “Darknet” was used in a 64-bit environment (Windows 10®, Microsoft Corp, Redmond WA, USA) with a single Graphics Processing Unit (GPU) on a board (NVIDIA Quadro P5000®, NVIDIA Corporation, Santa Clara CA, USA) which was equipped with 2560 compute unified device architecture (CUDA) cores and 16 GB of memory^39^.

#### Evaluation

We evaluated detection accuracy using the 150 original images, which were different from the training data. We manually counted all worms in respective evaluation data, a total of 5187 worms were included (mean ± standard deviation = 37 ± 14.2 per image, range: 3–86). Pre-processing of the original image was performed prior to evaluation. The DNN predicted the class and bounding box for each worm from the input image. A confidence score threshold, which is the probability that an anchor box contains the object, which would affect detection accuracy, was defined as 0.30 by selecting best detection accuracy using the validation data^40^.

Detection accuracy of the output bounding box was compared with the ground truth bounding box using precision, recall, and average precision (AP). These metrics were calculated using true-positive (TP), false-negative (FN), and false-positive (FP) instances^41^. Precision and recall measure the accuracy of predictions and how well objects are found, and are defined as follows:

2 $$Precision=\frac{TP}{TP+FP}$$

3 $$Recall=\frac{TP}{TP+FN}$$

The F1 score combines precision and recall into one metric by calculating the harmonic mean.

4 $$F1=\frac{2 \cdot Precision \times Recall}{Precision + Recall}$$

AP is a metric to measure the accuracy of object detection and is calculated for each class.

5 $$AP=\sum_{k=0}^{k=n-1}({Recall}_{k}-{Recall}_{k-1}){\cdot Precision}_{k}$$

**Figure 7:**
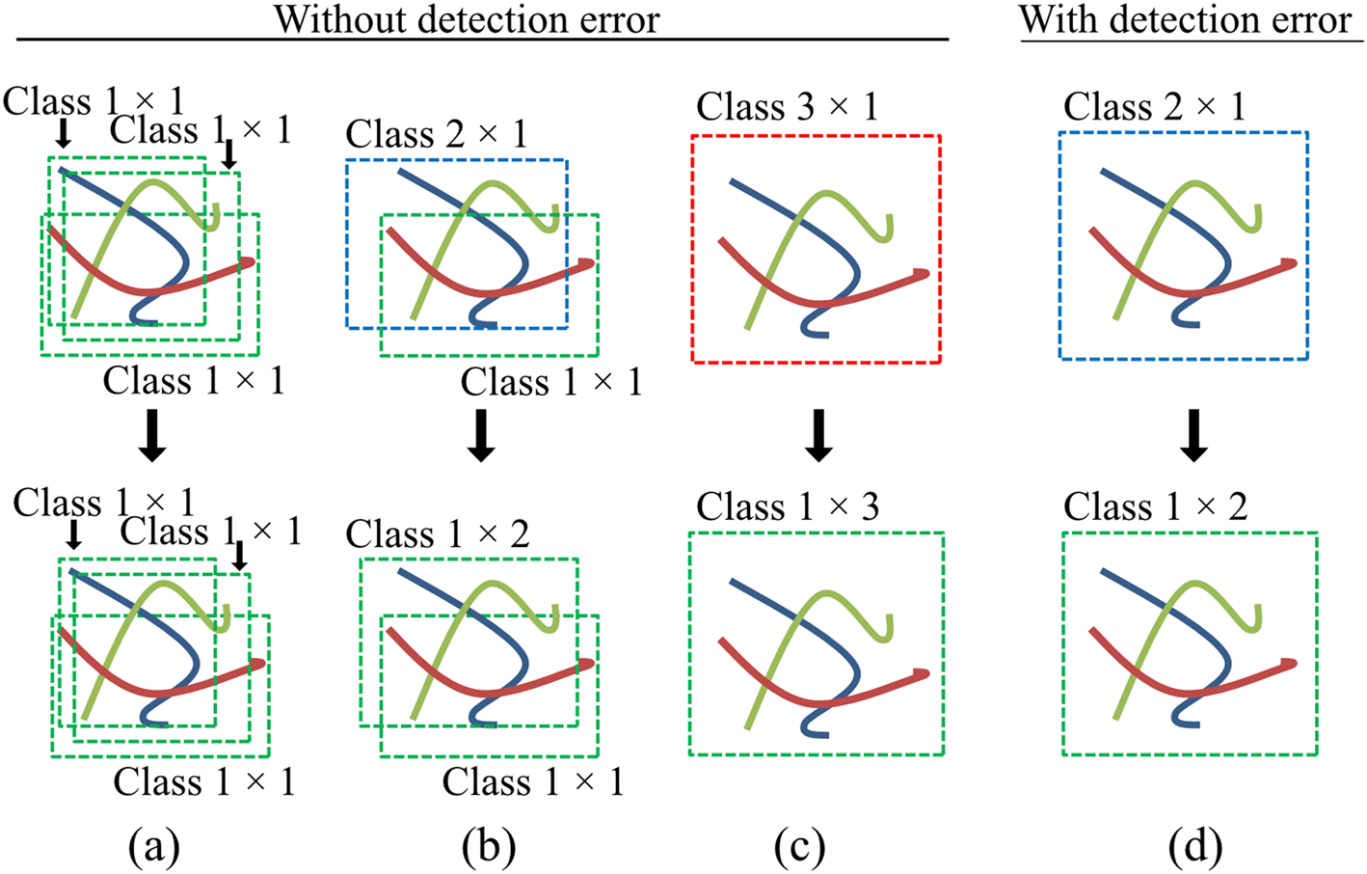
Examples of predicted classes for three worms with the multi-class classification (MCC). When the detection error is zero, (a) three worms are detected as (**a**) Class 1 × 1 (upper panel), and it is divided into Class1 × 1 (lower panel), this is same with the one-class classification (OCC), (**b**) Class 2 × 1 and Class 1 × 1 (upper panel), and it is divided into Class 1 × 3 (lower panel), (**c**) Class 3 × 1 (upper panel), and it is divided into Class1 × 3 (lower panel). (**d**) While the detection error is caused, Class 2 × 1 (upper panel), and it is divided into Class 1 × 2 (lower panel).

To compare evaluation metrics between the OCC and MCC, we evaluated the MCC as follows:

The total number of bounding boxes detected for the MCC might be smaller than that for the OCC, because the MCC detected 2–4 worms in a single bounding box. As an example using three worms, when the detection error was zero, the OCC detected 3 bounding boxes (Fig. 7a), while the MCC detected Class 1 × 3, Class 2 × 1 + Class 1 × 2, or Class 3 × 1 (upper panels in Fig. 7a–c). As a result, evaluation metrics would vary. If a detection error is caused by the DNN, the MCC would detect three worms as Class 2 × 1 (upper panel in Fig. 7d). This would be a false-positive case (zero true-positive) and could be an underestimation, although two worms were correctly detected. This would be recognized as 2 true-positives and 1 false-positive, hence our division of Class 2, Class 3, and Class 4 into Class 1 × 2, Class 1 × 3 and Class 1 × 4, respectively (lower panels in Fig. 7b and c).

## Data Availability

The data that support the findings of this study are available on request from the corresponding author but restrictions apply to the availability of these data, which were used under submitting to the patent, and so are not publicly available.
